# The Impact of Lighting Regimen and Feeding Program during Rearing on Hy-Line Brown Pullets at the End of Rearing and during Early Lay

**DOI:** 10.3390/ani14192850

**Published:** 2024-10-03

**Authors:** Wendy Isabelle Muir, Yeasmin Akter, Sebastian Kai Yi Kho, Kenneth Bruerton, Peter John Groves

**Affiliations:** 1 School of Life and Environmental Sciences, Faculty of Science, The University of Sydney, Camden, NSW 2570, Australia; yeasmin.akter@sydney.edu.au; 2 Sydney School of Veterinary Science, Faculty of Science, The University of Sydney, Camden, NSW 2570, Australia; skho8030@uni.sydney.edu.au (S.K.Y.K.); peter.groves@sydney.edu.au (P.J.G.); 3 Independent Researcher, Elanora, QLD 4221, Australia; kenb001@icloud.com

**Keywords:** feed restriction, reduced lighting, egg quality, egg production, body weight, feed intake, age of first egg, growth, photoperiod

## Abstract

**Simple Summary:**

The body weight of pullets (i.e., sexually immature chickens) immediately before they start to lay eggs can impact their subsequent egg production, feed intake, and feed efficiency. Therefore, pullets of a particular size may be required to meet performance targets throughout their egg-laying phase. This study evaluated lighting (hours of light/day) and feeding programs during rearing as tools to grow pullets to a designated size. Their performance was then monitored throughout their early egg-laying period. Compared to restricted feeding and shorter lighting/day, ad libitum (ad lib) feeding—or free access to feed—together with longer hours of light/day resulted in higher feed intake and heavier pullets. However, pullets fed ad lib but with fewer hours of light were the first to start to lay eggs. Interestingly, pullets reared with ad lib feeding continued to consume more feed throughout their early egg-laying period. They also produced more eggs, which were heavier than the eggs from hens that, as pullets, had been restricted to lower feed intake during rearing. Hence, lighting and feeding regimens during rearing are practical tools that can achieve specific pullet weight, feed intake, hen weight, egg production, and egg size throughout the early egg-laying period of commercial chickens.

**Abstract:**

As hen body weight (BW) impacts egg weight (EW) and feed efficiency, egg producers prefer pullets of a specific size to enter the egg-laying cycle. Lighting and feeding programs were tested to achieve target Hy-Line Brown pullet BW. Three feeding programs were implemented: ad libitum (ad lib); feeding to achieve breed standard weight for age (BSW); and feeding to achieve 88% BSW (managed). The feeding programs were used with either control lighting (CL: 10 h light/d from 7 weeks of age (WOA)) or reduced lighting (RL: 9 h light/d from 4 WOA). One-hundred and fifty pullets were assigned to each feeding program by lighting treatment during rearing. At 16 WOA, 70 pullets from each treatment during rearing were moved to cages and onto ad libitum feeding under a step-up photoperiod reaching 16 h light/d at 33 WOA. The age and weight of the first egg, hen BW, feed intake (FI), egg production (EP), and EW were measured until 36 WOA. At 16 WOA, pullets reared with ad lib feeding under CL had higher BW and cumulative FI (CFI) compared to ad lib feeding under RL. The latter were the earliest to lay, and the managed pullets under CL were the last to lay. Control lighting and BSW independently generated the heaviest first eggs. At 36 WOA, BW, EW, CFI, and cumulative egg production (CEP) were highest following ad lib feeding during rearing, while rearing under CL generated higher BW and EW but lower CEP than RL. Hence, lighting and feeding programs throughout rearing can regulate pullet growth, FI, and hen performance throughout early lay.

## 1. Introduction

Rearing of egg-laying pullets encompasses the time from their placement as day-old chicks in the rearing house until just prior to their sexual maturity, when they are transferred to the laying facility in preparation for the start of egg production (EP). Throughout the rearing period, all body components, including the muscles, gastrointestinal tract, reproductive organs, and skeletal system, develop [1]. The size of brown layer pullets at the end of the rearing phase is indicative of their ongoing growth trajectory throughout the laying phase [2,3]; that is, the heavier pullet at the end of rearing will continue as comparatively heavier, and the lighter pullet will remain at a comparatively lighter weight throughout lay. Hence, management of pullet feed intake (FI), growth, and body size during rearing may enable the establishment of these characteristics for the duration of the egg production cycle.

Currently, Australian egg producers prefer larger pullets at transfer to the laying house, as they tend to lay larger-sized eggs from the start of lay, also known as age of first egg (AFE), producing greater total egg mass (EM) at a younger age [2,4], compared to the smaller-sized hens. Larger-sized pullets also appear to manage the transition from the rearing to the laying facility more readily [5]. However, larger-sized hens consume more feed and have poorer feed efficiency throughout lay compared to smaller hens [6,7], which is a cost to the farming operation [5]. Their eggshell quality may also be compromised [6], and they may be more susceptible to fatty liver hemorrhagic syndrome (FLHS) [5,8,9]. Given the advantages and disadvantages of hens of different sizes, producers may prefer pullets of a specific size as they approach AFE. To achieve this, careful management of the flock throughout the rearing period is required. Practical on-farm options for managing pullet growth and size during rearing include the feeding program and lighting regimen, but these may also impact the age of sexual maturity, egg size, and EP [10].

With the aim to reduce the cost of feeding pullets, the impact of the quantity of feed consumed during rearing on pullet and hen performance was originally explored during the early stages of intensification of the egg industry [11,12]. Typically, pullets reared under restricted feeding had lower body weight (BW), lower FI, higher EP, lower feed conversion ratio (FCR), and were older when they produced their first egg [11,12]. In these original lines of egg-laying hens, the smaller birds produced fewer small eggs during early lay, with a higher peak rate of lay (ROL), a slower rate of decline in EP, and fewer bird mortalities throughout lay [12]. Compared to pullets fed ad libitum throughout rearing, feeding to 80% ad libitum FI resulted in 10–12% lower BW at the end of rearing and 2% lower BW at the end of the laying year [11]. More recent studies have also explored the management of FI during rearing on pullet growth rate and age of sexual maturity. These have included assessment with the White Leghorn [13], Babcock ISA White [14], and Rugao [15]. Most recently, Bahry et al. [16] managed the FI of White and Brown Lohmann birds from 8–25 weeks of age (WOA) to achieve 80% target BW, which also delayed their AFE compared to ad libitum-fed birds. However, the impact of managing FI during rearing on current Hy-Line Brown pullets at the end of rearing and their AFE and EP through peak lay has not been evaluated and is a focus of the current study.

The photoperiod/lighting regimen during rearing can also influence pullet size [4], age of sexual maturity [17,18], egg size, and the number of eggs produced throughout a laying cycle [4]. Typically, more hours of light, or a slower rate of decline in hours of light, during rearing facilitates higher FI, larger pullet size, a delay in sexual maturity, and larger eggs. However, the outcomes of studies exploring photoperiod have varied, most likely due to the strain of bird, different lighting regimens throughout rearing, and photostimulation used to initiate lay [19,20,21,22,23]. To determine the effect of the lighting program on Hy-Line Brown pullets, this study evaluated the impact of two lighting regimens during rearing on bird BW and cumulative FI to 16 WOA, AFE, and then hen BW, EP, and egg quality during early lay.

Additionally, there may be an interaction between the lighting and feeding programs used during rearing that could be employed to achieve a target BW at AFE. Typically, BW at sexual maturity is positively correlated with the hours of light and negatively associated with feed restriction during rearing [24]. However, the age of sexual maturity can vary, depending on the age of the pullets when the restrictions of feeding or lighting cease. Further, when used together, lighting and feeding programs can have an interactive effect on AFE [24]. In this study, lighting and feeding programs ceased when pullets were transferred to the laying facility at 16 WOA, which is reflective of typical age of transfer to the laying house in Australian egg production systems [25]. Hence, this study explored the impact of the feeding and lighting programs on pullet growth and AFE rather than the effect of the age at which the feeding and lighting programs were halted.

Therefore, to appraise management options to achieve a target pullet size at the end of rearing in Hy-Line Brown pullets, which are commonly used in the Australian egg- laying industry, this study evaluated the impact of two lighting regimens and three feeding programs throughout rearing on pullet FI, BW, development of the ovary and oviduct, and carcass composition at the end of rearing, when the pullets were 16 WOA. The effect of the lighting and feeding programs during rearing on pullet features at the end of rearing, including their FI, AFE, and corresponding EW and then hen BW, EP, FI, EW, and FCR through to 36 WOA were measured. Internal and external egg quality were assessed when hens were 32–33 WOA.

## 2. Materials and Methods

### 2.1. Ethical Approval

Hy-Line Brown grower pullets were reared until 16 WOA at the Zootechny Pty Ltd., research facility at Austral, NSW, Australia. All experimental procedures conducted during rearing were approved by the Birling Animal Ethics Committee (BAEC; Reference No. 1078). At 16 WOA, a subset of pullets was transported to the high-rise layer shed at the University of Sydney Poultry Research Unit, Camden, Australia, and all experimental procedures were approved by The University of Sydney Animal Ethics Committee (Protocol 2020/1827). All procedures were in accordance with the Australian code for the care and use of animals for scientific purposes [26].

### 2.2. Housing and Management of Pullets during Rearing

A total of 900 commercial Hy-Line Brown egg-layer pullets were obtained at the age of one day old. They had received infrared beak treatment and vaccination for infectious bronchitis and Marek’s disease virus at the hatchery (Specialised Breeders Australia, Bagshot, Victoria, Australia). Thirty pullets were placed in each of 30 floor pens (7 m^2^ floor space/pen). Each pen had one line of automatic nipple drinkers, manually filled feed hoppers that provided at least 15 cm/bird feeding space, and one wooden perch extending across the width of the pen opposite the entry point to the pen. Brooding was provided by gas-fired space heaters (Hired Hand^®^, Bremen, AL, USA) that were evenly positioned throughout the shed to meet the breed recommendations for temperature during rearing [1]. The floor pen facility had side curtains for ventilation and an insulated roof with foggers for heat control. The air temperature within the shed was 33 °C when the pullets were placed. The temperature was held from 30–33 °C for the first 3 days and then gradually reduced based on breed recommendations [1] and bird comfort to approximately 21 °C when pullets were 5 WOA. Temperature ranged from 18–25 °C for the remainder of the rearing period. Shed humidity was from 55–65% during the first week and 45–60% for the remainder of the rearing phase, with the exception of some afternoons when thunderstorm activity increased the humidity to 80–90%. The side curtains allowed for controlled lighting within the shed, and the shed lights were dimmable.

### 2.3. Experimental Design

**Figure 1:**
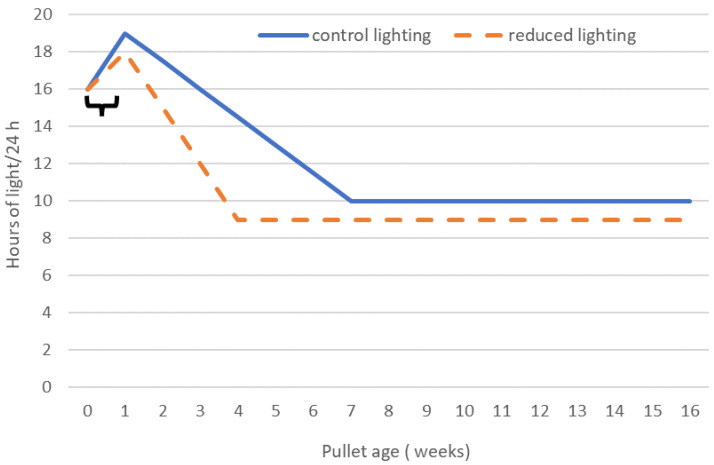
Total hours of light/24 h during rearing of Hy-line Brown pullets from placement until 16 weeks of age (WOA) under control lighting and reduced lighting regimens.

The study was a 2 × 3 factorial arrangement consisting of two lighting regimens and three feeding programs during rearing. A total of 30 day-old pullets allocated to each floor pen were weighed as a group at placement. During week 3, each pullet was identified using a uniquely numbered wing tag. Then, each pullet was individually weighed each week from 4 until 16 WOA. Bird uniformity within each floor pen was also calculated. The two ends of the shed were separated by a transverse lightproof curtain, allowing for two different, individually controlled lighting regimens to be run in each end of the shed. There were 15 floor pens in each end of the shed and for each lighting regimen. The control lighting (CL) regimen was generated by Hy-line^®^ (Hy-Line International, West Des Moines, IA, USA) based on the global location of the shed and scheduled to achieve 10 h light/24 h when birds were 7 WOA. The other end of the shed followed a reduced lighting (RL) regimen similar to the rapid lighting program of Hester et al. [23] and achieved 9 h light/24 h when birds were 4 WOA. The lighting regimens during rearing are presented graphically in Figure 1 and were as follows: CL regimen consisted of intermittent lighting of 4 h light:2 h dark/24 h from 0–1 WOA, 19 h light/24 h from 1–2 WOA, 17.5 h light/24 h from 2–3 WOA, 16 h light/24 h during 3–4 WOA, 14.5 h light/24 h during 4–5 WOA, 13 h light/24 h during 5–6 WOA, 11.5 h light/24 h during 6–7 WOA, and 10 h light/24 h from 7–16 WOA. In contrast, the RL regimen followed intermittent lighting of 4 h light: 2 h dark/24 h from 0–1 WOA, 18 h light/24 h from 1–2 WOA, 15 h light/24 h from 2–3 WOA, 12 h light/24 h from 3–4 WOA, and 9 h light/24 h from 4 to 16 WOA. Light intensity (lux) followed breed recommendation for age [1]. It ranged from 45–50 lux during the first week of rearing, then 20–25 lux until pullets were 4 WOA, when it was reduced to 5–10 lux. At 15 WOA, the light intensity was increased to 20–25 lux in preparation for pullet transfer to the layer facility. From placement until 4 WOA, all birds were fed ad libitum, and total FI for each floor pen of pullets was recorded. From 4 until 16 WOA, three feeding programs were allocated to 5 pens within each lighting regimen. To minimize bias, each group of 3 consecutive floor pens within each lighting regimen were set as a block, and the three feeding programs were allocated at random across the 3 consecutive floor pens. The feeding programs were as follows: 1, ad libitum feeding (identified as ad lib feeding); 2, Feeding-determined quantities of feed each day to achieve the breed standard weight (BSW) for age (identified as BSW feeding); and 3, feeding-determined quantities of feed each day to achieve 88% of the BSW for age (identified as managed feeding). Within each pen, 15 cm feed space was provided for each pullet, which exceeded the breed standard recommendation of 8 cm/pullet [1]. This ensured all birds were able to readily access the feed at the same time. Between 5 and 16 WOA, the uniformity of pullets within each floor pen ranged from 93.8 to 94.8%, which also exceeded the breed recommended uniformity of 85% or higher from 6 to 16 WOA [1].

Control lighting (CL): intermittent lighting 4 h light: 2 h dark from 0–1 weeks of age (WOA), 19 h light/24 h during 1–2 WOA, 17.5 h light/24 h during 2–3 WOA, 16 h light/24 h during 3–4 WOA, 14.5 h light/24 h during 4–5 WOA, 13 h light/24 h during 5–6 WOA, 11.5 h light/24 h during 6–7 WOA and 10 h light/24 h from 7–16 WOA.

Reduced lighting (RL): intermittent 4 h light: 2 h dark from 0–1 weeks of age (WOA), 18 h light/24 h from 1–2 WOA, 15 h light/24 h from 2–3 WOA, 12 h light/24 h from 3–4 WOA and 9 h light/24 h from 4–16 WOA.

Intermittent lighting of 4 h light: 2 h dark from 0–1 WOA.

Overall, there were six treatment groups of 150 pullets each, that is: CL with ad lib feeding, CL with BSW feeding, CL with managed feeding, RL with ad lib feeding, RL with BSW feeding, and RL with managed feeding. The weekly FI for each pen allocated to ad lib feeding from 4 to 16 WOA was determined by measuring total feed offered minus total feed remaining at the end of the week. The daily quantity of feed provided during the same one-week period to each floor pen of the BSW and managed feeding programs was calculated based on pullet BW compared to target BW for age and the quantity of feed provided during the previous week. The average daily FI/pullet each week for each of the six treatment groups from 4 to 16 WOA is presented in Table 1.

All birds received the same diets throughout the study. These were as follows: Barastoc^®^ starter crumble from chick placement until 5 WOA, then Barastoc^®^ grower crumble until pullets were 12 WOA (both from Ridley Agriproducts, Melbourne, Victoria, Australia), then developer mash until 16 WOA. The ingredient formulation and calculated nutrient composition of the developer diet are presented in Table 2 and Table 3, respectively. The diet was mixed in the feed mill at the Poultry Research Unit, The University of Sydney, Camden. Subsamples of each diet (starter, grower, and developer ration) were collected to determine gross energy (GE), crude protein (CP), crude fat (CF), calcium (Ca), and phosphorus (P) mineral composition, as described in detail by Muir et al. [5].

During rearing, all pullets were vaccinated against Newcastle disease at 2 WOA (Poulvac^®^ Newcastle live V4, Zoetis, Olot, Catalonia, Spain, batch no. 364389), fowl pox at 4 WOA (Nobilis^®^ Fowl Pox Vaccine, MSD Animal Health, Bendigo, Victoria, Australia, batch no. 2007205), infectious bronchitis at 5 WOA (Poulvac^®^ Bronchitis I, Zoetis, Charles City, IA, USA, batch no. 409873), infectious laryngotracheitis at 9 WOA (Poulvac^®^ Laryngo SA2, Zoetis, Charles City, IA, USA, batch no. 413185), avian encephalomyelitis at 11 WOA (Nobilis^®^ AEV vaccine, MSD Animal Health, Bendigo, Victoria, Australia, batch no. 1812806), Newcastle disease and egg drop syndrome at 14 WOA (Nobilis^®^ EDS + ND, MSD Animal Health, Bendigo, Victoria, Australia, batch no. G107A04), *Mycoplasma synoviae* and *Mycoplasma gallisepticum* at 14 WOA (Vaxsafe^®^ MS, Bioproperties Pty Ltd., Glenorie, New South Wales, Australia, batch no. MSH190822A and Vaxsafe^®^ MG, Bioproperties Pty Ltd., Glenorie, New South Wales, Australia, batch MGS191661B), and infectious bronchitis at 15 WOA (Poulvac^®^ Bronchitis A3, Zoetis, Charles City, IA, USA, batch no. 396843) following practices of using commercially available vaccines in Australia.

**Table 1:** Average daily feed intake of Hy-Line Brown pullets each week between 4 and 16 weeks of age.

Treatment		Average Feed Intake (g/Bird/Day)											
		Weeks of Age											
Lighting ^1^	Feeding ^4^	4–5	5–6	6–7	7–8	8–9	9–10	10–11	11–12	12–13	13–14	14–15	15–16 ^10^
CL ^2^	Ad lib ^5^	36.7	44.9	52.6	59.1	65.7	67.4	74.0	80.9	84.0	81.3	71.3	72.0
	SEM ^6^	0.26	0.11	0.42	0.86	1.47	2.06	1.24	1.37	1.69	2.39	2.31	0.91
CL	BSW ^7^	35.0	35.0	36.3	38.4	47.0	52.6	60.7	65.7	69.3	63.3	63.9	65.6
	SEM ^9^	0.00	0.00	0.00	0.00	0.00	0.00	0.00	0.00	0.00	0.00	0.00	0.00
CL	Managed ^8^	33.3	33.3	35.7	37.7	42.3	44.3	49.4	55.6	61.0	58.1	57.6	59.0
	SEM ^9^	0.00	0.00	0.00	0.00	0.00	0.00	0.00	0.00	0.00	0.00	0.00	0.00
RL ^3^	Ad lib	34.9	43.3	52.7	59.1	62.7	64.6	65.6	73.6	75.9	74.7	70.3	69.3
	SEM	0.44	0.89	0.61	0.72	1.19	0.78	1.30	2.04	1.76	1.56	0.92	0.75
RL	BSW	32.1	32.1	33.1	36.7	50.9	56.0	66.0	72.9	73.4	64.0	59.1	63.4
	SEM ^9^	0.00	0.00	0.00	0.00	0.00	0.00	0.00	0.00	0.00	0.00	0.00	0.00
RL	Managed	30.4	30.4	31.4	33.7	39.9	43.4	52.4	58.4	59.7	56.0	52.3	54.7
	SEM ^9^	0.00	0.00	0.00	0.00	0.00	0.00	0.00	0.00	0.00	0.00	0.00	0.00

^1^ Lighting: Lighting regimen during rearing, from chick placement until 16 weeks of age (WOA). ^2^ CL: Control lighting regimen, comprising intermittent lighting 4 h light:2 h dark/24 h from 0–1 WOA, 19 h light/24 h during 1–2 WOA, 17.5 h light/24 h during 2–3 WOA, 16 h light/24 h during 3–4 WOA, 14.5 h light/24 h during 4–5 WOA, 13 h light/24 h during 5–6 WOA, 11.5 h light/24 h during 6–7 WOA, and 10 h light/24 h from 7–16 WOA. ^3^ RL: Reduced lighting regimen comprising intermittent 4 h light:2 h dark/24 h from 0–1 WOA, 18 h light/24 h from 1–2 WOA, 15 h light/24 h from 2–3 WOA, 12 h light/24 h from 3–4 WOA, and 9 h light/24 h from 4–16 WOA. ^4^ Feeding: Feeding program between 4 and 16 WOA. ^5^ Ad lib: ad libitum feeding. ^6^ SEM: standard error of the mean. ^7^ BSW: Breed standard weight feeding to achieve pullet breed standard weight for age from 4–16 WOA. ^8^ Managed: Feeding to achieve 88% pullet breed standard weight for age between 4 and 16 WOA. ^9^ SEM: Standard error of the mean for pens with the same lighting by feeding treatment receiving the same amount of feed each week. ^10^ Cumulative feed intake from 4–16 WOA is presented in Table 4.

**Table 2:** Ingredients of Developer, Pre-lay, and Early-lay diets.

Ingredients (%)	Protein (%)	Developer (kg) (12–16 Weeks) ^6^	Protein (%)	Pre-lay (kg) (16–17.4 Weeks) ^7^	Protein (%)	Early-lay (kg) (17.5–36 Weeks) ^8^
Sorghum	10.00	240.15	8.40	250.00	10.80	245.00
Wheat	11.00	239.00	11.90	295.69	12.30	265.39
Barley	11.50	175.00	11.50	175.00	10.40	125.00
Millrun ^1^	15.00	165.00	-	-	-	-
Canola meal solvent	38.00	110.00	38.00	100.00	38.00	55.00
Soybean meal	46.50	34.00	46.50	102.00	47.30	170.00
Dicalcium phosphate		14.00		14.00		14.00
Limestone		9.00		25.00		20.00
Limestone grit (38%)		-		25.00		71.00
Soybean oil		5.00		5.00		25.00
Sodium bicarbonate		2.70		2.40		2.40
Lysine—HCl		1.65		1.15		1.20
Salt (NaCl)		1.20		1.60		1.70
Layer pre-mix ^2^		1.00		1.00		1.00
DL-methionine (conc)		0.80		1.10		2.25
Choline chloride (60%)		0.75		0.75		0.50
Avatec/Bovatec (20%) ^3^		0.50		-		-
AXTRA XB 201 ^4^		0.10		0.10		0.10
L-Threonine		0.10		0.15		0.40
AXTRAPHY TPT 100 ^5^		0.06		0.06		0.06
Total		1000.00		1000.00		1000.00

^1^ Millrun: byproduct of flour manufacturing consisting of ban, aleurone, germ, and pollard (Riverina Stock Feeds). ^2^ Layer premix composition/kg: Vitamin D3: 3.5 MIU; Vitamin A: 10 MIU; Vitamin E: 30 g; Vitamin K3: 3 g; Vitamin B1: 2.5 g; Vitamin B2: 5.5 g; Vitamin B3: 30 g; Vitamin B5: 9 g; Vitamin B6: 4 g; Vitamin B12: 0.2 g; Biotin H: 0.15 g; Copper: 8 g; Iodine: 1.5 g; Selenium: 0.25 g; Iron: 50 g; Zinc: 60 g; Manganese: 60 g; Carophyll Red 10%: 3.1 g; Carophyll Yellow 10%: 2.9 g; Ethoxyquin: 75 g. ^3^ Zoetis Avatec-Bovatec contains Lasalocid sodium 900 g/kg (90%). ^4^ AXTRA XB 201 contains xylanase and beta glucanase. ^5^ AXTRAPHY TPT 100 contains phytase enzyme. ^6^ Developer diet was fed to all pullets from 12 to 16 weeks of age. ^7^ Pre-lay diet was fed to all birds from 16 to 17.4 weeks of age. ^8^ Early-lay diet was fed to all birds from 17.5 to 36 weeks of age.

**Table 3:** Calculated and analyzed nutrient composition of Developer, Pre-lay, and Early-lay diets.

Nutrients	Developer Diet (12–16 Weeks) ^1^	Pre-lay Diet (16–17.4 Weeks) ^2^	Early-lay Diet (17.5–36 Weeks) ^3^
*Calculated value*			
ME-enzyme (MJ/kg)	11.41	11.6	11.73
NE layer (MJ/kg)	8.72	8.87	11.73
Crude protein (%)	15.57	16.09	17.62
Lysine (%)	0.76	0.80	0.90
Methionine (%)	0.33	0.37	0.49
Methionine & cystine (%)	0.65	0.68	0.80
Threonine (%)	0.55	0.59	0.66
Isoleucine (%)	0.56	0.62	0.70
Leucine (%)	1.18	1.24	1.41
Tryptophan (%)	0.20	0.21	0.22
Arginine (%)	0.82	0.90	1.02
Stand. ileal digest Lys. (%)	0.67	0.73	0.82
Crude fat (%)	2.69	2.39	4.19
Linoleic acid (%)	1.43	1.21	2.21
Total xanthophylls (mg/kg)	6.00	6.00	6.00
Red xanthophylls (mg/kg)	3.10	3.10	3.10
Yellow xanthophyl (mg/kg)	2.90	2.90	2.90
Ash (%)	5.20	9.51	13.5
Calcium (%)	1.00	2.55	4.09
Available phosphorus	0.45	0.45	0.45
Total phosphorus (%)	0.75	0.64	0.61
Sodium (%)	0.18	0.18	0.18
Chloride (%)	0.18	0.18	0.18
Choline (mg/kg)	1297.70	1365.50	1406.90
Lasalocid^®^ (mg/kg)	100.00	-	-
*Analyzed value*			
Gross energy (MJ/kg)	16.35	15.80	13.90
Crude protein (%)	16.00	14.50	17.40
Crude fat (%)	3.00	2.40	4.00
Calcium (%)	0.82	2.00	4.09
Phosphorus (%)	0.69	0.60	0.65

^1^ Developer diet was fed to all pullets from 12 to 16 weeks of age. ^2^ Pre-lay diet was fed to all pullets from 16 to 17.4 weeks of age. ^3^ Early-lay diet was fed to all birds from 17.5 to 36 weeks of age.

**Table 4:** Average body weight at 4, 12, and 16 weeks of age (WOA) and feed intake from 0–4 and 4–16 WOA of Hy-Line Brown pullets.

Treatment		BW ^9^ (g) 4 WOA ^10,11^	BW (kg) 12 WOA	BW (kg) 16 WOA	Cumulative	Cumulative
Lighting ^1^	Feeding ^5^				FI ^12^/Bird (g) 0–4 WOA ^11^	FI/Bird (kg) 4–16 WOA
CL ^2^	Ad lib ^6^	318.0	1.25 ^A^	1.52 ^A^	513	5.53 ^A^
CL	BSW ^7^	315.9	1.10 ^C^	1.37 ^C^	511	4.43
CL	Managed ^8^	314.5	0.95 ^E^	1.21 ^D^	506	3.97
RL ^3^	Ad lib	305.7	1.23 ^B^	1.48 ^B^	492	5.23 ^B^
RL	BSW	303.1	1.09 ^C^	1.37 ^C^	489	4.48
RL	Managed	310.1	1.00 ^D^	1.23 ^D^	503	3.80
SEM ^4^		4.0	0.006	0.01	10	-
Main effects						
Lighting	CL	316.1	1.10	1.37	510	4.63
	RL	306.3	1.10	1.36	494	4.50
	SEM	2.3	0.003	0.004	5	0.11
Feeding ^5^	Ad lib	311.8	1.24	1.50	502	5.40
	BSW	309.5	1.10	1.37	500	4.47
	Managed	312.3	0.97	1.22	504	3.87
	SEM	2.9	0.004	0.01	8	0.21
*p*-Value						
	Lighting	<0.001	0.669	0.078	0.057	-
	Feeding	0.294	<0.001	<0.001	0.316	-
	Lighting × Feeding	0.086	<0.001	<0.001	0.657	0.007

^1^ Lighting: Lighting regimen during rearing, from chick placement until 16 weeks of age (WOA). ^2^ CL: Control lighting regimen comprising intermittent lighting 4 h light:2 h dark/24 h from 0–1 WOA, 19 h light/24 h during 1–2 WOA, 17.5 h light/24 h during 2–3 WOA, 16 h light/24 h during 3–4 WOA, 14.5 h light/24 h during 4–5 WOA, 13 h light/24 h during 5–6 WOA, 11.5 h light/24 h during 6–7 WOA, and 10 h light/24 h from 7–16 WOA. ^3^ RL: Reduced lighting regimen comprising intermittent 4 h light:2 h dark/24 h from 0–1 WOA, 18 h light/24 h from 1–2 WOA, 15 h light/24 h from 2–3 WOA, 12 h light/24 h from 3–4 WOA, and 9 h light/24 h from 4–16 WOA. ^4^ SEM: standard error of the mean. ^5^ Feeding: Feeding program between 4 and 16 WOA. ^6^ Ad lib: ad libitum feeding. ^7^ BSW: Breed standard weight feeding to achieve pullet breed standard weight for age from 4–16 WOA. ^8^ Managed: Feeding to achieve 88% pullet breed standard weight for age between 4 and 16 WOA. ^9^ BW: body weight. ^10^ WOA: weeks of age. ^11^ 4 WOA: when pullets were 4 weeks of age and prior to the introduction of the feeding programs. ^12^ FI: feed intake. ^A–E^ Means within a column with different superscripts differ at *p* < 0.05.

### 2.4. Carcass, Organ, and Femur Characteristics at 16 Weeks of Age

At 16 WOA, 15 pullets from each of the six treatments, consisting of three pullets from each treatment floor pen, were selected at random to assess carcass composition, liver, and bone health. Each bird was weighed and then humanely euthanized. The skin over the breast was retracted, and the breast muscle scored (range 0–3: 0 being very lean with little breast muscle, and 3 being substantial breast muscle) [1]. The curvature of the keel bone was assessed on a 4-point scale ranging from straight keel (score 1) to mild (score 2), moderate (score 3), or severe (score 4) curvature [27]. The liver was evaluated for FLHS, where scores ranged from 0–5, as described by Shini et al. [8]. Development of the reproductive organs was evaluated by measuring the length of the oviduct using a ruler (cm) and the width (mm) of the largest ovum using a digital Vernier caliper with an accuracy of ±0.01 mm. The abdominal fat pad and liver were excised and weighed, and their weight was expressed as a percentage of pullet BW.

The right femur was collected, frozen, and stored at −20 °C. In preparation for analysis, the femur was thawed to room temperature, and the skin, ligaments, and muscles were removed. The femur was then weighed using a digital scale. The length of the femur and its external width at its mid-length was measured. Traditional bone density indicators of bone weight to length (femur index: g/mm) [28], expressed as a percent, were calculated, where a higher weight to length index is indicative of higher bone density. The femur was then assessed for breaking strength (N), taken as the peak force to fracture the bone mid-shaft (horizontal plane) using a texture analyzer (Perten TVT 6700, Stockholm, Sweden) fitted with a break probe (671,170 break probe with a 675,045-break rig set) [5]. The cortical thickness and medullary bone diameter were measured at the breaking point using digital Vernier calipers with an accuracy of ±0.01 mm. The broken bones were used to determine their ash content [5]. The dry weight of the femur was measured following 24 h at 105 °C, and the ash weight was expressed as a percent of the dry weight.

### 2.5. Hen Management from 16 to 36 Weeks of Age

At 16 WOA, 14 birds from each of the five floor pen replicates of each treatment, i.e., 70 birds from each of the six light × feeding rearing treatments (420 birds in total), were transferred to the layer housing facility at the Poultry Research Unit, The University of Sydney, Camden. To identify the pullets to be transferred, all pullets from each treatment were listed in numerical order based on their wing tag number and then selected at random using a random number generator. At the layer facility, each bird was housed in an individual pen (dimensions 25 × 50 × 50 cm) with an individual feeder to allow for precise measurement of individual hen feed consumption, egg production, and calculation of FCR. From 16 WOA, all birds were held under the same lighting regimen, which provided 11 h light/24 h at 16 WOA, reaching 16 h light/24 h at 33 WOA, where it was held until hens were 36 WOA. Light intensity was 20–25 lux.

From 16 WOA, all birds were fed ad libitum with a mash diet. A pre-lay diet was provided from 16 to 17.4 (i.e., 17 weeks and 4 days) WOA followed by an early-lay diet from 17.5 to 36 WOA. Subsamples of each diet were collected for analysis of GE, CP, CF, and Ca and P mineral composition, as previously described. The formulation and calculated nutrient composition of the pre-lay and early-lay diets are presented in Table 2 and Table 3 respectively.

### 2.6. Hen Body Weight and Egg Production Performance

Each hen was individually weighed at 17, 18, 19, and 36 WOA. Individual hen FI was also measured for each week, and cumulative FI was calculated from 17.5 to 36 WOA by measuring feed offered minus feed remaining. The AFE for each hen was recorded, and the average weight of her first three eggs was calculated. Total egg production was recorded for each hen until 36 WOA. Eggs were collected each day and weighed using a digital scale, and the average EW/week was determined for each hen. Weekly hen EP was calculated from the number of eggs laid per hen across the 7-day period. Average daily EM for each hen was calculated as EP expressed as a percent, multiplied by the average EW (g) for that week. The FCR was calculated on a weekly basis for each hen as FI g/EM g. Cumulative FI, EP, EM, and FCR for each hen and treatment group was then determined from when they were placed onto the early-lay diet at 17.5 WOA until they were 36 WOA.

### 2.7. Egg Quality Assessment

Twelve focal birds were chosen at random from each of the six treatment groups, and their eggs were collected at 32 and 33 WOA to assess egg quality. Egg quality assessment was limited to these two weeks due to COVID-19-related restrictions imposed by the New South Wales state government. The eggs were collected on two days each week, and they were assessed the day they were collected. The first assessment each week involved measuring egg dimensions, internal egg quality, and eggshell thickness. Each egg was weighed, and its height (from top to base) and width (at the egg equator) were measured using a digital caliper. Egg shape index was calculated as egg width/egg length × 100 [29]. The egg was then broken out onto a flat glass surface, and the albumen height was measured using an albumen height gauge TSS (Technical Services and Supplies, York, UK). Yolk color was determined using a DSM^®^ Yolk Color Fan (color range 1–15: 1 being very pale yellow and 15 being darker orange). Haugh unit (HU) values were calculated using the formula: 100 × log (h − 1.7 × w^0.37^ + 7.6), where h = albumen height (mm) and w = egg weight (g) [30]. Each eggshell (with shell membrane intact) was gently rinsed and then dried at room temperature for three days. Once dried, the shell weight was measured using a digital scale to an accuracy of 0.01 g and expressed relative to the EW. Eggshell thickness was the average of measurements taken at the top, equator, and base of the egg using a digital caliper. The second egg collected from each focal bird each week was used to assess eggshell breaking strength (N) at the broad end of the egg. This entailed a 3-point bending test of the peak force to fracture using a texture analyzer (Perten TVT 6700, Stockholm, Sweden) fitted with a cylindrical probe 75 mm in diameter.

### 2.8. Statistical Analysis

Continuous data were analyzed in a factorial design comprising the 2 lighting regimens × 3 feeding programs for each observation using the generalized linear model procedure of STATISTICA Version 6 [31]. The interaction between the factors and their main effects were assessed. The data are presented in tabular format following this arrangement. Means were separated using the Tukey-honestly significant difference model. All data are presented as means ± pooled standard error of the mean (SEM). The probability value that denotes statistical significance is *p* < 0.05. Categorical data from scoring assessments was subjected to Kruskal–Wallis analysis, suitable for non-parametric data, also using STATISTICA.

## 3. Results

### 3.1. Diet Composition

The Barastoc^®^ starter diet consisted of 16.2 MJ/kg GE, 21.8% CP, 2.5% CF, 1.19% Ca, and 0.55% P. The Barastoc^®^ grower diet contained 16.2 MJ/kg GE, 17% CP, 2.5% CF, 0.87% Ca, and 0.61% P. As shown in Table 3, the developer diet consisted of 16.35 MJ/kg GE, 16% CP, 3% CF, 0.82% Ca, and 0.69% P.

### 3.2. Pullet Body Weight and Feed Intake from Placement to 16 Weeks of Age

**Figure 2:**
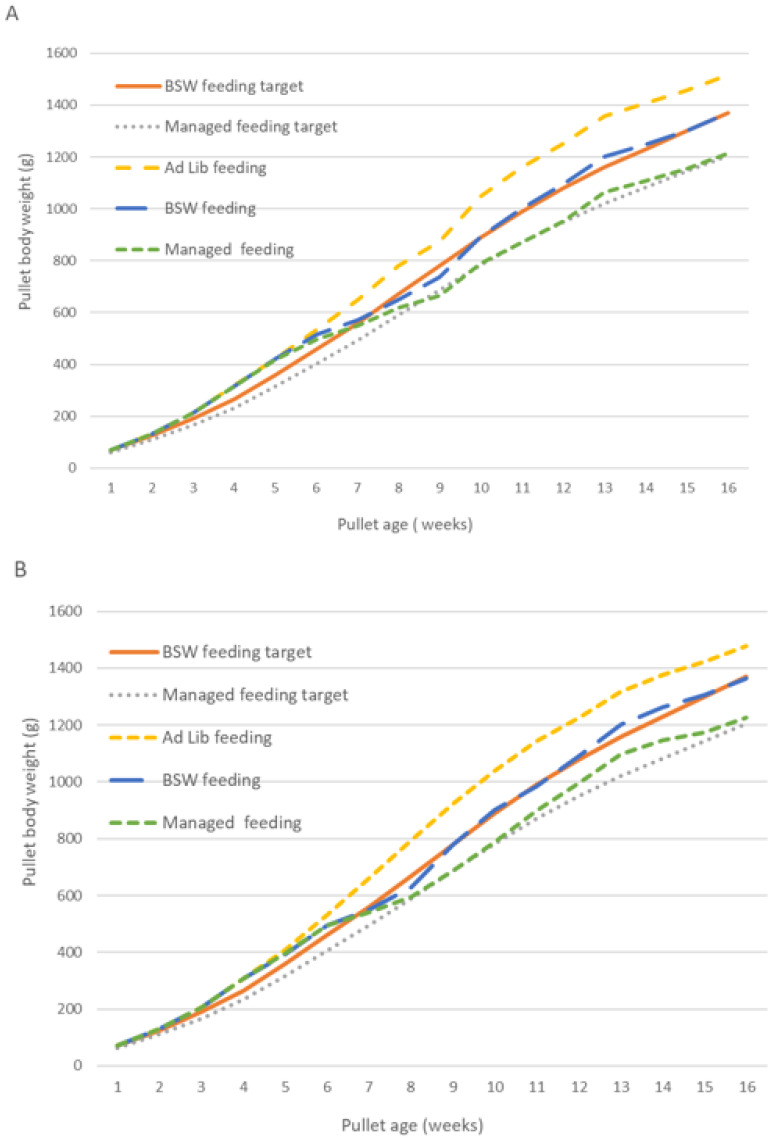
Hy-Line Brown pullet growth during rearing, from 1–16 weeks of age, under control lighting (CL) (**A**) and reduced lighting (RL) (**B**) regimens with feeding programs ad lib feeding, feeding to breed standard weight (BSW feeding) for age, and feeding to 88% BSW for age (managed feeding) from 4–16 weeks of age. The target weights for age for BSW and managed feeding are included. (**A**) Control lighting (CL): intermittent lighting 4 h light:2 h dark from 0–1 weeks of age (WOA), 19 h light during 1–2 WOA, 17.5 h light during 2–3 WOA, 16 h light during 3–4 WOA, 14.5 h light during 4–5 WOA, 13 h light during 5–6 WOA, 11.5 h light during 6–7 WOA, and 10 h from 7–16 WOA. (**B**) Reduced lighting (RL): intermittent 4 h light:2 h dark from 0–1 weeks of age (WOA), 18 h light from 1–2 WOA, 15 h light from 2–3 WOA, 12 h light from 3–4 WOA, and 9 h light from 4–16 WOA.

The average pullet weight for the three feeding programs under the two lighting regimens compared to target weight for the BSW feeding and managed feeding programs are presented from 1–16 WOA in Figure 2 (A CL and B RL). Pullet BW at 4, 12, and 16 WOA are presented in Table 4, together with cumulative FI from 0 to 4 and 4 to 16 WOA. There were no differences in BW when pullets were placed in the floor pens at day-old age. The lighting regimen resulted in birds of different average weight at 4 WOA, which was prior to the introduction of the feeding programs. The heavier pullets had been exposed to CL, whereas the lighter pullets were under the RL regimen (316.1 vs. 306.3 g, respectively; *p* < 0.001; Table 4). Under both lighting regimens, average pullet BW at 4 WOA was higher than the recommended breed standard weight for age of 260–270 g [1].

From placement until 4 WOA, the average FI of pullets under CL was approaching significance (*p* = 0.057) compared to the RL regimen (510 g/b vs. 494 g/b, respectively; Table 4). At 12 WOA, average BW was influenced by an interaction (*p* < 0.001) between the lighting regimen and feeding program. Pullets reared under CL on ad lib feeding were the heaviest (1.25 kg), followed by birds on ad lib feeding under RL (1.23 kg; Table 4). The lightest birds had been reared under CL with managed feeding (0.95 kg). Similarly, average BW at 16 WOA was also affected by the interaction of the lighting regimen and feeding program (*p* < 0.001). Again, the pullets that had been fed ad lib under CL were the heaviest (1.52 kg), followed by birds fed ad lib under RL (1.48 kg). At 16 WOA, the ad lib-fed birds of both lighting regimens were heavier than Hy-Line Brown recommended BW for age of 1.33–1.41 kg [1]. At 16 WOA, the lightest birds under both lighting regimens had been on the managed feeding program from 4 to 16 WOA, with average BW of 1.21 kg from CL and 1.23 kg with RL (Table 4; Figure 2A,B). As the target BW for the managed feeding program at 16 WOA was 1.17–1.24 kg (88% of BSW for age), this was achieved with both lighting regimens. Pullets fed to meet BSW of 1.33–1.41 kg at 16 WOA also met that target, averaging 1.37 kg BW under both lighting regimens (Table 4; Figure 2A,B). Cumulative FI from 4 to 16 WOA could only be statistically analyzed for the ad lib-fed birds, as the FI of the BSW and managed feeding programs had been regulated by the research team as dictated by the experimental design. Of the ad lib-fed pullets, those that had been reared under the CL regimen consumed more feed compared to pullets reared under RL (5.53 kg vs. 5.23 kg, respectively; *p* = 0.007; Table 4).

### 3.3. Pullet Carcass, Organ, and Femur Characteristics at 16 Weeks of Age

Table 5 presents the carcass and organ traits of the pullets at 16 WOA. The feeding programs generated differences in breast score, keel curvature score, ovum width, and oviduct length. Pullets from ad lib feeding had the highest measures for each of these parameters, and managed feeding the lowest (*p* < 0.05). The BSW feeding program achieved breast and keel curvature scores that were similar to the ad lib-fed pullets (*p* > 0.05) and similar ovum width and oviduct length to pullets reared within the managed feeding program. The lighting regimen during rearing did not alter any of the carcass or organ features.

When pullets were 16 weeks old, ad lib feeding during rearing increased the relative weight of the abdominal fat pad compared to both the BSW and managed feeding programs (1.76%, 0.42%, and 0.21%, respectively; *p* < 0.001; Table 5). In addition, the pullets assigned to the BSW and the managed feeding programs had heavier relative liver weight at 16 WOA compared to ad lib-fed pullets (1.24%, 1.29%, and 1.05%, respectively; *p* < 0.001). All FLHS scores were 0, with no evidence of hemorrhage. 

At 16 WOA, the lighting regimen during rearing had no effect on fresh and dry femur weight, femur length, width, femur index (femur weight to length index), bone marrow diameter, cortical thickness, femur bone ash (percent), or femur breaking strength (*p* > 0.05; Table 6). In contrast, the feeding program during rearing did influence some characteristics of the femur. The fresh and dry femur weight in ad lib-fed birds was heavier than in pullets from the BSW and managed feeding programs (*p* < 0.001), with pullets from the managed feeding program having the lightest femoral weight. The femur of ad lib-fed birds was longer than that of the managed feeding program (87.3 cm vs. 84.9 cm, respectively; *p* < 0.001) and wider (8.23 mm) than pullets from both the BSW and managed feeding programs (7.85 and 7.72 mm, respectively; *p* < 0.001). There was no difference (*p* > 0.05) in the femur width of pullets fed to achieve BSW, nor those from the managed feeding program. The femur index was highest in ad lib-fed birds (10.3%), intermediate in pullets of BSW feeding (9.94%), and lowest in pullets reared with the managed feeding program (9.37%; *p* < 0.001). At 16 WOA, the diameter of the femur bone marrow was wider in the ad lib-fed birds (7.26 mm; *p* < 0.001) compared to both BSW and managed feeding treatments (6.89 and 6.76 mm, respectively), the latter two being similar to each other. The relative weight of femur ash was highest (*p* = 0.038) in the BSW-fed birds (33.2%) and lowest (32.1%) in pullets of the ad lib feeding program, with managed feeding (32.4%) being the same as both the ad lib and BSW feeding programs. Cortical thickness and femur breaking strength at 16 WOA were not affected (*p* > 0.05) by the feeding program.

### 3.4. Performance Following Transfer to the Laying House

From 17 to 19 WOA, the lighting regimen during rearing did not affect bird BW nor FI, but the feeding program during rearing generated differences in BW at 17–19 WOA (Table 7). Overall, pullets on the ad lib feeding program during rearing remained heavier than those from both the BSW and managed feeding programs, and birds from the BSW feeding program remained heavier than birds of the managed feeding program (*p* < 0.001). However, during their first week with ad libitum feeding in the laying house—that is, 16–17 WOA—pullets that had been on the managed feeding program during rearing consumed more feed (62.9 g/d; *p* < 0.001) than birds from the BSW (55.7 g/d) and ad lib (48.5 g/d) feeding programs during rearing. Additionally, birds reared with the BSW feeding program also consumed more feed from 16–17 WOA than birds reared with ad lib feeding (Table 7). Despite this, at 17 WOA, pullets that had been fed ad lib during rearing remained heavier than pullets from the BSW and managed feeding programs. During 17–18 WOA, there were no differences in FI of pullets due to the feeding programs during rearing (*p* = 0.166), and average BW at 18 WOA remained as ad lib-fed birds being heavier (1.66 kg) than both BSW (1.53 kg) and managed feeding (1.42 kg), and the BSW-fed birds were heavier than birds of the managed feeding program (*p* < 0.001). Between weeks 18 and 19, birds from the BSW feeding program during rearing had the highest FI (97.4 g/d), which was higher (*p* < 0.001) than birds from the ad lib feeding (91.3 g/d) and managed feeding (93.3 g/d) programs during rearing. At 19 WOA, ad lib-fed birds were still the heaviest, weighing 1.78 kg, and the birds from managed feeding program were the lightest at 1.55 kg (*p* < 0.001). The pullets from the BSW feeding program during rearing were of intermediate weight (1.66 kg) at 19 WOA.

The lighting and feeding treatments during rearing interacted to affect AFE (*p* = 0·038; Table 7). Pullets reared under RL and fed ad lib were the first to lay eggs at 19.16 WOA, while CL with ad lib feeding resulted in the second youngest AFE of 19.53 WOA. The latter was not different from the AFE for pullets reared under RL with managed feeding or RL with feeding to BSW (19.73 and 19.67 WOA, respectively). Combining CL with BSW feeding resulted in first egg at 19.86 WOA, which was not different from the latter two groups. Control lighting with the managed feeding program generated the oldest AFE at 20.33 WOA. The average weight of the first three eggs was independently affected by the lighting and feeding treatments during rearing (Table 7). The first three eggs from pullets reared under CL were heavier than those of pullets reared under RL (49.5 g vs. 48.3 g, respectively; *p* = 0.007). Within the feeding programs, BSW feeding generated the heaviest first three eggs, averaging 49.5 g, being heavier than the first eggs of pullets reared with ad lib feeding (48.0 g; *p* = 0.021). The first three eggs produced by pullets reared under the managed feeding program were of intermediate weight (49.2 g), which was not different from the EW of pullets under the ad lib and BSW feeding programs.

### 3.5. Hen Performance through 36 Weeks of Age

Hen BW, EP, and EW at 36 WOA, cumulative FI, cumulative EP, cumulative EM, and cumulative FCR from 17.5–36 WOA are presented in Table 8. At 36 WOA, hens that had been reared under the CL regimen were heavier than those from RL (2.13 kg vs. 2.07 kg; *p* < 0.001). Ad lib feeding during rearing generated the heaviest (2.17 kg) hens compared to BSW feeding (2.12 kg) and managed feeding hens (2.01 kg; *p* < 0.001). There were no differences in EP during week 36. Average EW was higher in hens reared under the CL regimen compared to RL (63.3 g vs. 62.4 g; *p* = 0.015). Of the feeding programs during rearing, the heaviest eggs were produced by hens from ad lib feeding (63.3 g), and the lightest eggs were from birds from managed feeding (62.7 g; *p* = 0.029), with the EW of hens from BSW feeding (63.1 g) not differing from either of the other two feeding programs.

Cumulative FI from 17.5–36 WOA was higher (*p* = 0.012) in hens from the ad lib feeding program (15.4 kg) compared to managed feeding during rearing (15.1 kg), neither of which differed from feeding to BSW during rearing (15.3 kg; Table 8). Both the lighting regimens and feeding programs during rearing independently influenced cumulative EP from 17.5–36 WOA. Birds from RL produced more eggs compared to the CL regimen (113 vs. 110 eggs; *p* < 0.001), and hens from ad lib feeding during rearing had the highest average cumulative EP compared to the BSW and managed feeding programs (114 vs. 111 vs. 109 eggs respectively; *p* < 0.001). The higher cumulative EP and EW of hens from ad lib and BSW feeding programs during rearing increased their cumulative EM at 36 WOA (6.63 and 6.50 kg, respectively) compared to managed feeding (6.30 kg; *p* < 0.001). The lighting and feeding treatments during rearing did not affect (*p* > 0.05) cumulative FCR from 17.5 to 36 WOA (Table 8).

### 3.6. Egg Quality at 32–33 Weeks of Age

The external and internal egg quality characteristics at 32 and 33 WOA are presented in Table 9. The lighting and feeding treatments during rearing had no effect (*p* > 0.05) on EW, nor on egg shape index, yolk color, relative shell weight, shell thickness, or shell breaking strength. However, the relative eggshell weight was approaching significance (*p* = 0.061) between the RL and CL regimens (10.6% vs. 10.3%, respectively). Haugh unit experienced an interaction between the feeding and lighting treatments during rearing (*p* = 0.040). The managed feeding program under both CL and RL resulted in the highest HU (108.2 HU and 106.7 HU, respectively), and eggs with the lowest HU (97.9 HU) were produced by hens reared with ad lib feeding under CL.

## 4. Discussion

Maintaining BW is a vital objective in hen production systems, as hens that are above the breed recommendation produce eggs of lower albumen quality [32] and are prone to being obese [6]. The BW trajectory of hens throughout the laying phase is established by the start of lay [3,7]. Therefore, to achieve a predetermined BW at the start of lay and an expected BW trajectory throughout lay, close supervision of pullet growth using management practices such as lighting regimen and feeding program is required during rearing. Hence, this study evaluated the effect of lighting regimen and feeding programs on pullet growth, reproductive organ development, carcass composition, liver health, and bone quality at the end of rearing. Following the transfer of the pullets to the layer shed, a gradual increase in photoperiod, and ad libitum feeding for all birds, their initial FI, BW, AFE, and average EW were recorded. Moreover, EP, FI, and FCR were measured from 17.5 to 36 WOA, and egg quality was assessed at 32–33 WOA.

Management of the lighting and feeding programs during rearing has been found to influence BW before the start of lay, including level of body fat deposition and regulation of sexual maturity [21,24,33]. In this study, lighting alone and the number of h light/24 h impacted pullet growth rate and BW by 4 WOA, which was prior to the introduction of the feeding programs. Pullets held under longer day length in CL (16 h light/24 h at 3–4 WOA) were on average 10 g heavier at 4 WOA than pullets housed under RL (12 h light/24 h at 3–4 WOA). Further, and in agreement with Lewis et al. [20] and Lera [4], the longer period of light/24 h with CL allowed for higher FI compared to the fewer h of light/24 h under RL. At 12 and 16 WOA, h of light did not impact BW alone but in conjunction with the feeding program. Specifically, the ad lib feeding program with CL generated the heaviest pullets, and, as dictated by the experimental design, the lightest pullets were on managed feeding under both lighting regimens. Following the study plan, the latter had also consumed less feed than the ad lib and feeding to BSW programs in order to achieve their target BW for age. Of the pullets within the ad lib feeding program, those reared with CL had more time to eat, and between 4 and 16 WOA, each pullet had consumed an average 300 g more than the pullets reared under RL. This also matched with an expected heavier BW [20], where at 16 WOA the former was on average 40 g heavier than pullets of ad lib feeding under RL (Table 4). A similar difference in the BW (45 g) of Hy-Line Brown pullets at 17 WOA was also identified following their rearing under a slow compared to a rapid step-down lighting regimen [22].

The growth curves illustrated in Figure 2A,B show pullet BW following a similar upward trajectory after the initial adjustment to the amount of feed offered in each of the three feeding programs that were introduced when the pullets were 4 WOA. Interestingly, during the second, third, and fourth weeks on the developer diet (i.e., 13–16 WOA), the average daily FI of pullets on the ad lib feeding programs was lower than during the first week on the developer diet (12–13 WOA; Table 1). Some of this reduction in FI is most likely due to the pullets adapting to the developer diet, which was in mash form compared to the crumble form of the Barastoc^®^ starter and grower diets. Concurrently, the amount of feed provided to the BSW and managed feeding programs under both lighting regimens was less during the final three weeks on the developer diet, as their BW had moved above the target weight at the start of week 13 (Figure 2A,B). Critically, despite lower FI in all feeding programs, pullet BW continued to increase over this time.

Of the feeding treatments, pullets from the managed feeding program during rearing had the lowest breast muscle score (1.57 out of a possible 3) at 16 WOA, and the highest score was reported for the ad lib-fed birds (1.97; Table 5). Rearing under RL with ad lib feeding was the only treatment that generated a breast muscle score of 2, which is the score recommended by the breed standards [1]. However, the lower breast muscle content of lighter pullets from the managed feeding program during rearing may be indicative of their lower daily energy requirement for maintenance compared to heavier hens. This could facilitate a relatively higher amount of energy available for growth and, once in lay, EP [34] compared to the ad lib-fed birds. However, at 36 WOA, the cumulative EP of lighter hens from managed feeding during rearing was lower than the hens fed ad lib during rearing. It should be noted that total EP is likely to have been impacted by the later AFE of birds on managed feeding during rearing, which resulted in fewer days to produce eggs compared to the ad lib-fed pullets. The effect of AFE on cumulative EP may even out when considered across a longer laying cycle. Hence, the ongoing impact of hen BW on EP requires longer term evaluation; for example, until hens are 72 WOA or older. Additionally, the relative size of the abdominal fat pad was greatly reduced with feeding to BSW or managed feeding when compared to ad lib feeding (i.e., 0.42%, 0.21%, and 1.76% relative weight, respectively; Table 5). This is the likely outcome of fewer abdominal adipose cells following feed restriction and hence a smaller fat pad, as identified in broiler chicks [35].

At the end of rearing, ad lib-fed pullets had the lowest relative liver weight compared to the pullets fed to achieve BSW for age or 88% BSW for age under the managed feeding program (Table 5). When exposed to feed restrictions, a higher relative liver weight has been attributed to an adaptation to the limited quantity of feed [36], which may have also occurred with these pullets.

At 16 WOA, pullets of the ad lib and BSW feeding programs had the highest keel bone curvature scores (Table 5). While pullets from both of these feeding programs were heavier than the pullets from the managed feeding program, a direct association between hen BW and keel deformities has not been previously reported [37,38]. Typically, keel damage occurs when the bird collides with other birds or structures or when they fall from a height [39]. Interestingly, studies involving earlier strains of chickens [40] found keel damage most often occurred before sexual maturity and was related to the perching environment. In the current study, the perching environment was similar for all treatments, with one perch positioned at the same height, towards the back of the pen, away from the feeding tubes. It had been expected that pullets on the managed feeding program, where competition for feed was highest, would have had higher keel bone damage and therefore a higher keel curvature score, but that was not observed. Multiple factors can lead to keel bone injuries [41,42], with housing complexity most frequently associated with bone abnormality. But, as previously mentioned, the rearing pens used in this study were not complex, housing few structures, and they were similar to each other. Hence, the reasons for the higher level of keel curvature in birds from the ad lib and BSW compared to the managed feeding program in this study are not immediately evident.

Femur characteristics of 16 WOA pullets, including weight, length, width, and percent ash, strongly aligned with their feeding program and hence their comparative BW, the latter agreeing with the findings of Muir et al. [3,43]. However, there was no difference in femur bone breaking strength. Similarly, no differences in femur bone breaking strength were observed between heavier and lighter ISA Brown hens at either 70 or 90 WOA, despite differences in their femur weight, length, and index [3,43]. In contrast, Kraus et al. [44] compared three genotypes of egg-laying hens, and the heaviest strain (Dominant Partridge D300), which also had the longest, widest, and heaviest femur, was also the most resilient to bone fractures when compared to the lighter Czech Golden Spotted and White Leghorn strains.

Irrespective of the lighting regimen, pullets with ad lib access to feed during rearing illustrated more advanced reproductive maturation at 16 WOA, as was evident through their highest average ovum width and longest oviduct compared to pullets of the BSW and managed feeding programs (Table 5). These findings corroborate with others, where pullets reared with ad lib feeding are typically heavier and are likely to reach sexual maturity at an earlier age than pullets on restricted FI during rearing [11,12,45]. Concurring with this study, Lu et al. [15] also reported slower pullet growth and delayed sexual maturity, as determined through less apparent ovarian follicular development and a shorter oviduct, following restricted feeding. Further, compared to pullets reared with restricted feeding, heavier ad lib-fed pullets also had more advanced body development for age, including breast muscle and a higher relative abdominal fat pad (Table 5), the latter also being reported by Johnson et al. [46].

All pullets experienced photostimulation on transfer to the layer shed, where the initial photoperiod was 11 h/24 h. This was a 2 h increase in photoperiod/24 h for pullets reared under RL compared to a 1 h increase for pullets that had been reared under CL. The comparatively greater increase in photoperiod of pullets from RL during rearing is likely to have contributed to their earlier start of lay [47]. However, it should be noted that lighting interacted with feeding on AFE in this study (Table 7). Additionally, the effect of changing from either a comparatively longer or shorter photoperiod on the induction of lay may vary with the strain of hen. For example, compared to rearing under longer day length (10 or 12 h), Lohmann White pullets reared with shorter day length (6 or 8 h) were older at 50% egg production following photostimulation (12.5 h light) at 18 WOA, whereas rearing day length had no effect on the age of 50% lay in Lohmann Brown pullets [20].

The interactive effect of lighting and feeding during rearing on AFE observed in this study (Table 7) has also been reported by others [24,48]. The heavier hens from ad lib feeding during rearing were the first to lay, and this was earliest with the RL (19.16 WOA), who experienced the greatest increase in photoperiod on transfer to the layer shed compared to CL (19.53 WOA). The oldest pullets at AFE (20.3 WOA) had been reared with the combined managed feeding and CL treatments. The 8-d difference between the earliest and latest AFE reflects the amalgamation of the extremes of heavier pullets with greater photostimulation in the former and lighter pullets with a smaller photostimulation in the latter.

Generally, the later the AFE, the heavier the weight of the first eggs [14,17,45,49]. Similarly, in this study, earlier AFE with ad lib feeding during rearing generated lighter initial EW (48 g) compared to 49.5 and 49.2 g EW from hens that had been reared with the BSW and managed feeding programs, respectively, and were older with their first egg (Table 7). Consequently, in an egg market that rewards heavier eggs or greater EM, there may be a tendency to delay sexual maturity and the start of lay [19]. Alternatively, if the number of eggs produced is more critical than egg size, photostimulation is likely to be scheduled at an earlier age when pullets are at lower BW [47] but with an aim to increase EW as quickly as possible thereafter.

Once transferred to the layer house, all birds were held under the same step-up lighting schedule with ad libitum provision of a common diet. During the first week in the layer shed—that is, at 16–17 WOA—the ranking for ADFI of birds in the three feeding programs during rearing (Table 1) was reversed (Table 7); that is, pullets from the managed feeding program during rearing had the highest ADFI and ad lib feeding program the lowest ADFI. This concurs with the findings of Berg and Bearse [48] and Gous et al. [24], who also noted that the longer the period of controlled FI during rearing, the higher the increase in FI during the first week of ad libitum feeding. In this study, restrictions to feed quantity were for the same period, i.e., from 4–16 WOA, but the more restricted the feed quantity, i.e., the managed feeding program, the higher the ADFI during the first week of ad libitum feeding. However, despite this, the ranking of hen BW at 17, 18, and 19 WOA remained as it had been during rearing, i.e., hens from ad lib feeding during rearing remained the heaviest, and those from managed feeding during rearing were the lightest. It should be noted that during the first few weeks in the layer house, the BW of pullets from the BSW feeding program under both lighting regimens during rearing moved to the upper range of the BSW for age; i.e., 1.66 kg at 19 WOA, compared to the recommended 1.57–1.67 kg for age [1]. Further, by 17 WOA, the BW of pullets from the managed feeding program (1.45 kg) was considerably higher than the target 88% of BSW for age of 1.27 kg. Hence, pullets reared with restricted FI experienced a notable increase in BW once on ad libitum feeding in individual cages.

The rapid increase in ADFI by all pullets between 18 and 19 WOA (Table 7) reflects the increasing demands of rapid ovarian development [50] and body growth as they approach the start of lay [51]. From 17.5 to 36 WOA (Table 8), CFI was similar for both lighting regimens during rearing but was lower in hens reared with managed feeding compared to ad lib feeding. This concurs with Lee et al. [11] and Balnave [12], indicating that ad lib feeding during rearing establishes a habit of higher FI that persists in the laying phase. This leads to ongoing heavier BW and a tendency for hens to become obese [6]. Although once on ad libitum feeding in the layer house, hens reared with restricted FI also increased in BW to be above their target BW for age, they continued to consume less with sustained lower BW compared to the hens reared on ad lib feeding (Table 8). Hence, controlling FI during rearing established the habit of comparatively lower FI, which continued into lay and moderated BW gain compared to hens that were fed ad lib during rearing.

Even though the lighting regimen and feeding program during rearing did not alter EP at 36 WOA, the hens that had been reared under RL had higher cumulative EP from 17.5 to 36 WOA compared to rearing under the CL regimen (Table 8). The earlier AFE under RL provided more laying days and therefore more eggs, but, as reported by Morris [19], the eggs were smaller at both the start of lay (Table 7) and at 36 WOA (Table 8). Hens reared with ad lib feeding had higher cumulative EP between 17.5 and 36 WOA compared to hens reared with feeding to BSW and managed feeding (Table 8), which is also a consequence of their earlier AFE. Akbaş and Takma [52] concluded that, compared to BW and EW, age at sexual maturity was the most effective at increasing EP. As previously discussed, in the current study, the youngest pullets at first egg had been on ad lib feeding during rearing and were the heaviest. In contrast, Balnave [12] found restricted feeding during rearing generated higher EP as the result of higher peak ROL and a slower decline in EP. However, egg production can vary with bird strain [16], which may have contributed to the higher EP observed with the earlier strains of hens following feed restriction during rearing [11,12] as opposed to the higher EP with ad lib feeding during rearing of current egg-laying hens.

At 36 WOA, EW was independently affected by the lighting and feeding treatments during rearing. The heavier eggs were from hens reared under CL or ad lib feeding, which were also heavier birds, and, in the case of CL, were older at first egg. These findings also concur with the outcomes of earlier studies [17,45,49,53]. In contrast, ad lib-fed pullets produced their first eggs at a younger age, which were smaller compared to the first eggs from pullets reared with managed feeding. However, at 36 WOA, hens reared with ad lib feeding had maintained their heavier BW but were now producing heavier eggs compared to the hens reared with lower FI on managed feeding.

Between 17.5 and 36 WOA, hens reared with ad lib feeding and feeding to BSW produced higher cumulative EM compared to hens that had been reared with the managed feeding program. The rearing treatments of lighting and feeding did not influence FCR at 36 WOA nor the cumulative FCR between 17.5 and 36 WOA. This disagrees with the higher FCR of hens reared on ad libitum feeding [54,55]. Further, heavier hens are frequently reported to have higher FCR; for example, from 18–50 WOA [5].

Several features of the egg, including HU, eggshell weight, and eggshell strength, are indicative of egg quality [56]. Haugh units, a reflection of albumen quality, were impacted by the interaction of lighting and feeding during rearing, being highest at 32–33 in hens that had been reared with managed feeding under both lighting regimens (Table 9). As observed by others [6,57], controlling hen FI and BW gain facilitates eggs with superior albumen quality. While the percent shell weight tended to be lower in the larger eggs produced by hens reared under CL, it remained above 9.5%, the critical point below which there is an increased risk of eggshell cracks and fractures [58]. Taken together with similar shell strength in the eggs of hens reared with the different treatments, shell quality was not affected by the lighting or feeding treatments during rearing.

## 5. Conclusions

Pullet BW can be manipulated through the lighting regimen and the feeding program during rearing. Ad lib feeding and the CL regimen generated the heaviest pullets at the end of rearing and when hens were 36 WOA. Control lighting also resulted in a delay in the start of lay and therefore lower total EP through to 36 WOA, but the eggs were heavier than those of hens reared with RL. Compared to ad lib feeding, managing feed allocation for lower feed consumption during rearing reduced pullet BW, development and growth trajectory, and established a habit of lower FI and comparably lower BW throughout early lay. Further, hens reared with lower FI compared to ad lib feeding during rearing started to lay eggs at an older age, and their first eggs were heavier. But, at 36 WOA, they had produced fewer eggs, which were now smaller but of superior albumen quality than the eggs of hens reared with ad lib feeding. Feeding and lighting during rearing did not impact eggshell quality in early lay. To gain an accurate account of the impact of the feeding and lighting programs during rearing on EP, the production cycle should extend until hens are 70 plus WOA. A longer laying period would also enable assessment of the impact of these rearing conditions in achieving target pullet weight at the start of rearing on hen health, feed efficiency, and egg quality later in the laying cycle.

## Figures and Tables

**Table 5:** Breast score, keel curvature, relative abdominal fat pad and liver weight, ovum width, and oviduct length of 16-week-old Hy-Line Brown pullets.

Treatment		Breast Score ^9^ (0–3)	Keel Curvature ^10^ (1–4)	Abdominal Fat Pad Weight ^11^ (%)	Liver Weight ^12^ (%)	Ovum Width ^13^ (mm)	Oviduct Length ^14^ (cm)
Lighting ^1^	Feeding ^5^						
CL ^2^	Ad lib ^6^	1.93	1.67	1.71	1.04	4.00	11.0
CL	BSW ^7^	1.93	1.47	0.40	1.27	2.67	7.5
CL	Managed ^8^	1.53	1.27	0.21	1.33	2.40	7.5
RL ^3^	Ad lib	2.00	1.53	1.81	1.06	3.67	12.5
RL	BSW	1.93	1.67	0.43	1.21	3.27	8.6
RL	Managed	1.60	1.27	0.20	1.25	2.00	7.7
SEM ^4^		0.12	0.13	0.11	0.03	0.36	0.85
Main effects							
Lighting	CL	1.80	1.47	0.77	1.21	2.89	8.67
	RL	1.84	1.49	0.81	1.17	2.98	9.60
	SEM	0.07	0.08	0.07	0.02	0.21	0.50
Feeding	Ad lib	1.97 ^a^	1.60 ^a^	1.76 ^a^	1.05 ^b^	3.83 ^a^	11.7 ^a^
	BSW	1.93 ^a^	1.57 ^a^	0.42 ^b^	1.24 ^a^	2.77 ^b^	8.1 ^b^
	Managed	1.57 ^b^	1.27 ^b^	0.21 ^b^	1.29 ^a^	2.20 ^b^	7.6 ^b^
	SEM	0.09	0.09	0.08	0.02	0.25	0.60
*p*-Value							
	Lighting	0.651	0.837	0.667	0.136	0.763	0.187
	Feeding	<0.01	0.025	<0.001	<0.001	<0.001	<0.001
	Lighting × Feeding	0.949	0.450	0.871	0.271	0.096	0.771

^1^ Lighting: Lighting regimen during rearing, from chick placement until 16 weeks of age (WOA). ^2^ CL: Control lighting regimen comprising intermittent lighting 4 h light:2 h dark/24 h from 0–1 WOA, 19 h light/24 h during 1–2 WOA, 17.5 h light/24 h during 2–3 WOA, 16 h light/24 h during 3–4 WOA, 14.5 h light/24 h during 4–5 WOA, 13 h light/24 h during 5–6 WOA, 11.5 h light/24 h during 6–7 WOA, and 10 h light/24 h from 7–16 WOA. ^3^ RL: Reduced lighting regimen comprising intermittent 4 h light:2 h dark/24 h from 0–1 WOA, 18 h light/24 h from 1–2 WOA, 15 h light/24 h from 2–3 WOA, 12 h light/24 h from 3–4 WOA, and 9 h light/24 h from 4–16 WOA. ^4^ SEM: standard error of the mean. ^5^ Feeding: Feeding program between 4 and 16 WOA. ^6^ Ad lib: ad libitum feeding. ^7^ BSW: Breed standard weight feeding to achieve pullet breed standard weight for age from 4–16 WOA. ^8^ Managed: Feeding to achieve 88% pullet breed standard weight for age between 4 and 16 WOA. ^9^ Breast score: based on 4-point scale [1]. ^10^ Keel curvature: based on 4-point scale [27]. ^11^ Abdominal fat pad weight (%): fat pad weight as a percent of pullet live body weight. ^12^ Liver weight (%): liver weight as a percent of pullet live body weight. ^13^ Ovum width: width of the largest ovum. ^14^ Oviduct length: length of the oviduct from the anterior infundibulum to the cloaca. ^a,b^ Means within a column with different superscripts differ at *p* < 0.05.

**Table 6:** Characteristics of the femur bone of 16-week-old Hy-Line Brown pullets.

Treatment		Fresh Weight (g)	Dry Weight (g)	Length (mm)	Width (mm)	W:L Index ^9^	Bone Marrow Diameter (mm)	Cortical Thickness (mm)	Ash ^10^ (%)	Breaking Strength (N) ^11^
Lighting ^1^	Feeding ^5^									
CL ^2^	Ad lib ^6^	9.09	6.24	87.9	8.20	10.34	7.24	0.966	31.8	162.1
CL	BSW ^7^	8.69	5.55	86.6	7.83	10.04	6.86	0.971	33.6	166.5
CL	Managed ^8^	7.82	5.00	84.8	7.73	9.23	6.76	0.965	32.4	169.9
RL ^3^	Ad lib	8.90	6.01	86.8	8.25	10.26	7.28	0.965	32.4	160.4
RL	BSW	8.41	5.44	85.4	7.88	9.85	6.92	0.958	32.9	161.9
RL	Managed	8.10	5.21	85.0	7.71	9.52	6.75	0.964	32.4	167.8
SEM ^4^		0.17	0.13	0.60	0.10	0.16	0.10	0.005	0.46	4.70
Main effects										
Lighting	CL	8.53	5.60	86.4	7.92	9.87	6.95	0.968	32.6	166.2
	RL	8.47	5.55	85.7	7.95	9.88	6.98	0.962	32.5	163.3
	SEM	0.10	0.08	0.35	0.01	0.09	0.06	0.003	0.26	2.65
Feeding	Ad lib	8.99 ^a^	6.13 ^a^	87.3 ^a^	8.23 ^a^	10.30 ^a^	7.26 ^a^	0.966	32.1 ^b^	161.3
	BSW	8.55 ^b^	5.50 ^b^	86.0 ^ab^	7.85 ^b^	9.94 ^b^	6.89 ^b^	0.965	33.2 ^a^	164.2
	Managed	7.96 ^c^	5.10 ^c^	84.9 ^b^	7.72 ^b^	9.37 ^c^	6.76 ^b^	0.965	32.4 ^ab^	168.9
	SEM	0.12	0.09	0.42	0.07	0.11	0.07	0.004	0.32	3.34
*p*-Value										
	Lighting	0.657	0.691	0.151	0.73	0.942	0.682	0.151	0.874	0.466
	Feeding	<0.01	0.025	<0.001	<0.001	<0.001	<0.001	0.997	0.038	0.260
	Lighting×Feeding	0.211	0.229	0.423	0.93	0.299	0.916	0.424	0.384	0.942

^1^ Lighting: Lighting regimen during rearing, from chick placement until 16 weeks of age (WOA). ^2^ CL: Control lighting regimen comprising intermittent lighting 4 h light:2 h dark/24 h from 0–1 WOA, 19 h light/24 h during 1–2 WOA, 17.5 h light/24 h during 2–3 WOA, 16 h light/24 h during 3–4 WOA, 14.5 h light/24 h during 4–5 WOA, 13 h light/24 h during 5–6 WOA, 11.5 h light/24 h during 6–7 WOA, and 10 h light/24 h from 7–16 WOA. ^3^ RL: Reduced lighting regimen comprising intermittent 4 h light:2 h dark/24 h from 0–1 WOA, 18 h light/24 h from 1–2 WOA, 15 h light/24 h from 2–3 WOA, 12 h light/24 h from 3–4 WOA, and 9 h light/24 h from 4–16 WOA. ^4^ SEM: standard error of the mean. ^5^ Feeding: Feeding program between 4 and 16 WOA. ^6^ Ad lib: ad libitum feeding. ^7^ BSW: Breed standard weight feeding to achieve pullet breed standard weight for age from 4–16 WOA. ^8^ Managed: Feeding to achieve 88% pullet breed standard weight for age between 4 and 16 WOA. ^9^ Femur W:L index: standardized femur weight: length index based on 100 g/mm. ^10^ Femur ash (%): femur ash weight as a percent of dry femur weight. ^11^ N: Newton. ^a–c^ Means within a column with different superscripts differ at *p* < 0.05.

**Table 7:** Hy-Line Brown pullet body weight and feed intake during the first three weeks after transfer to the layer shed, average age of first egg, and average weight of the first three eggs.

Treatment		BW ^9^ (kg) 17 WOA ^10^	BW (kg) 18 WOA	BW (kg) 19 WOA	FI ^11^ (g/d) 16–17 WOA	FI (g/d) 17–18 WOA	FI (g/d) 18–19 WOA	Age First Egg (wks)	Weight
Lighting ^1^	Feeding ^5^								First 3 Eggs (g)
CL ^2^	Ad lib ^6^	1.56	1.67	1.79	48.8	51.4	91.0	19.53 ^C^	48.5
CL	BSW ^7^	1.48	1.54	1.67	57.2	53.7	97.1	19.86 ^B^	50.0
CL	Managed ^8^	1.44	1.43	1.54	62.4	54.2	90.8	20.33 ^A^	50.0
RL ^3^	Ad lib	1.55	1.65	1.76	48.2	53.8	91.5	19.16 ^D^	47.6
RL	BSW	1.49	1.51	1.66	54.2	53.7	97.7	19.67 ^BC^	49.0
RL	Managed	1.46	1.42	1.56	63.3	55.2	95.9	19.73 ^BC^	48.4
SEM ^4^		0.01	0.01	0.01	9.33	7.69	1.41	0.09	0.50
Main effects									
Lighting	CL	1.49	1.54	1.66	56.1	53.1	93.0	19.91	49.5
	RL	1.49	1.53	1.66	55.2	54.3	95.0	19.52	48.3
	SEM	0.01	0.01	0.01	5.39	4.44	0.81	0.04	0.30
Feeding	Ad lib	1.55 ^a^	1.66 ^a^	1.78 ^a^	48.5 ^c^	52.6	91.3 ^b^	19.35	48.0 ^b^
	BSW	1.48 ^b^	1.53 ^b^	1.66 ^b^	55.7 ^b^	53.7	97.4 ^a^	19.77	49.5 ^a^
	Managed	1.45 ^c^	1.42 ^c^	1.55 ^c^	62.9 ^a^	54.7	93.3 ^b^	20.03	49.2 ^ab^
	SEM	0.01	0.01	0.01	6.00	5.44	1.00	0.06	0.40
*p*-Value									
	Lighting	0.554	0.115	1.00	0.386	0.188	0.078	<0.001	0.007
	Feeding	<0.001	<0.001	<0.001	<0.001	0.166	<0.001	<0.001	0.021
	Lighting×Feeding	0.313	0.618	0.092	0.350	0.566	0.201	<0.038	0.793

^1^ Lighting: Lighting regimen during rearing, from chick placement until 16 weeks of age (WOA). ^2^ CL: Control lighting regimen comprising intermittent lighting 4 h light:2 h dark/24 h from 0–1 WOA, 19 h light/24 h during 1–2 WOA, 17.5 h light/24 h during 2–3 WOA, 16 h light/24 h during 3–4 WOA, 14.5 h light/24 h during 4–5 WOA, 13 h light/24 h during 5–6 WOA, 11.5 h light/24 h during 6–7 WOA, and 10 h light/24 h from 7–16 WOA. ^3^ RL: Reduced lighting regimen comprising intermittent 4 h light:2 h dark/24 h from 0–1 WOA, 18 h light/24 h from 1–2 WOA, 15 h light/24 h from 2–3 WOA, 12 h light/24 h from 3–4 WOA, and 9 h light/24 h from 4–16 WOA. ^4^ SEM: standard error of the mean. ^5^ Feeding: Feeding program between 4 and 16 WOA. ^6^ Ad lib: ad libitum feeding. ^7^ BSW: Breed standard weight feeding to achieve pullet breed standard weight for age from 4–16 WOA. ^8^ Managed: Feeding to achieve 88% pullet breed standard weight for age between 4 and 16 WOA. ^9^ BW: body weight. ^10^ WOA: weeks of age. ^11^ FI: feed intake. ^A–D^ Means within a column with different superscripts differ at *p* < 0.05. ^a–c^ Means within a column with different superscripts differ at *p* < 0.05.

**Table 8:** Production performance of Hy-Line Brown hens from 17.5 to 36 weeks of age.

	Treatment	BW ^9^ (kg) 36 WOA ^10^	Cum. ^11^ FI ^12^ (kg) /Hen 17.5–36 WOA	Egg Production Eggs/Hen 36 WOA	Cum. Egg Production/ Hen 17.5–36 WOA	Egg Weight (g/d) 36 WOA	Cum. Egg Mass/Hen (kg) 17.5–36 WOA	Cum. FCR ^13^ (g Feed/g Egg Mass)/Hen 17.5–36 WOA
Lighting ^1^	Feeding ^5^							
CL ^2^	Ad lib ^6^	2.22	15.5	6.89	114	63.7	6.63	2.34
CL	BSW ^7^	2.14	15.3	6.84	110	63.5	6.45	2.40
CL	Managed ^8^	2.04	15.1	6.94	107	62.8	6.24	2.42
RL ^3^	Ad lib	2.13	15.2	6.81	115	63.0	6.64	2.39
RL	BSW	2.11	15.3	6.88	112	62.7	6.56	2.34
RL	Managed	1.98	15.1	6.87	111	61.6	6.35	2.39
SEM ^4^		0.19	0.10	0.09	0.8	0.46	0.06	0.03
Main effects								
Lighting	CL	2.13	15.3	6.90	110	63.3	6.44	2.39
	RL	2.07	15.2	6.85	113	62.4	6.52	2.36
	SEM	0.01	0.06	0.05	0.5	0.27	0.04	0.02
Feeding	Ad lib	2.17 ^a^	15.4 ^a^	6.85	114 ^a^	63.3 ^a^	6.63 ^a^	2.34
	BSW	2.12 ^b^	15.3 ^ab^	6.86	111 ^b^	63.1 ^ab^	6.50 ^a^	2.37
	Managed	2.01 ^c^	15.1 ^b^	6.91	109 ^c^	62.7 ^b^	6.30 ^b^	2.40
	SEM	0.01	0.01	0.06	0.6	0.33	0.04	0.02
*p*-Value								
	Lighting	<0.001	0.309	0.427	<0.001	0.015	0.131	0.229
	Feeding	<0.001	0.012	0.605	<0.001	0.029	<0.001	0.094
	Lighting×Feeding	0.386	0.510	0.503	0.081	0.846	0.674	0.644

^1^ Lighting: Lighting regimen during rearing, from chick placement until 16 weeks of age (WOA). ^2^ CL: Control lighting regimen comprising intermittent lighting 4 h light:2 h dark/24 h from 0–1 WOA, 19 h light/24 h during 1–2 WOA, 17.5 h light/24 h during 2–3 WOA, 16 h light/24 h during 3–4 WOA, 14.5 h light/24 h during 4–5 WOA, 13 h light/24 h during 5–6 WOA, 11.5 h light/24 h during 6–7 WOA, and 10 h light/24 h from 7–16 WOA. ^3^ RL: Reduced lighting regimen comprising intermittent 4 h light:2 h dark/24 h from 0–1 WOA, 18 h light/24 h from 1–2 WOA, 15 h light/24 h from 2–3 WOA, 12 h light/24 h from 3–4 WOA, and 9 h light/24 h from 4–16 WOA. ^4^ SEM: standard error of the mean. ^5^ Feeding: Feeding program between 4 and 16 WOA. ^6^ Ad lib: ad libitum feeding. ^7^ BSW: Breed standard weight feeding to achieve pullet breed standard weight for age from 4–16 WOA. ^8^ Managed: Feeding to achieve 88% pullet breed standard weight for age between 4 and 16 WOA. ^9^ BW: body weight. ^10^ WOA: weeks of age. ^11^ Cum: cumulative. ^12^ FI: feed intake. ^13^ FCR: feed conversion ratio. ^a–c^ Means within a column with different superscripts differ at *p* < 0.05.

**Table 9:** Internal egg and eggshell quality between 32 and 33 weeks of age of eggs of Hy-Line Brown hens.

Treatment		Egg Weight (g)	Egg Shape Index ^9^ (%)	Haugh Unit (HU)	Yolk Color Score ^10^ (1–15)	Shell Weight ^11^ (%)	Shell Thickness (mm)	Shell Breaking Strength (N) ^12^
Lighting ^1^	Feeding ^5^							
CL ^2^	Ad lib ^6^	62.9	77.9	97.9 ^C^	11.7	10.2	0.392	45.1
CL	BSW ^7^	63.4	78.4	99.5 ^B^	11.5	10.2	0.406	49.2
CL	Managed ^8^	60.8	78.8	108.2 ^A^	11.5	10.5	0.401	48.0
RL ^3^	Ad lib	60.5	78.5	101.1 ^BC^	11.8	10.6	0.405	50.6
RL	BSW	62.1	78.8	104.2 ^AB^	11.8	10.5	0.409	49.7
RL	Managed	60.4	80.5	106.7 ^A^	11.7	10.6	0.405	47.9
SEM ^4^		1.13	0.64	1.24	0.15	0.19	0.01	2.20
Main effects								
Lighting	CL	62.4	78.4	101.9	11.6	10.3	0.400	47.5
	RL	61.0	79.2	104.0	11.7	10.6	0.407	49.3
	SEM	0.65	0.37	0.71	0.08	0.11	0.004	1.3
Feeding	Ad lib	61.7	78.2	99.4	11.7	10.4	0.399	47.9
	BSW	62.8	78.6	101.8	11.7	10.3	0.408	49.4
	Managed	60.6	79.6	107.5	11.6	10.6	0.403	47.9
	SEM	0.80	0.46	0.88	0.10	0.13	0.01	1.5
*p*-Value								
	Lighting	0.135	0.098	0.040	0.138	0.061	0.264	0.271
	Feeding	0.171	0.074	<0.001	0.550	0.507	0.495	0.693
	Lighting×Feeding	0.617	0.537	0.041	0.795	0.711	0.760	0.377

^1^ Lighting: Lighting regimen during rearing, from chick placement until 16 weeks of age (WOA). ^2^ CL: Control lighting regimen comprising intermittent lighting 4 h light:2 h dark/24 h from 0–1 WOA, 19 h light/24 h during 1–2 WOA, 17.5 h light/24 h during 2–3 WOA, 16 h light/24 h during 3–4 WOA, 14.5 h light/24 h during 4–5 WOA, 13 h light/24 h during 5–6 WOA, 11.5 h light/24 h during 6–7 WOA, and 10 h light/24 h from 7–16 WOA. ^3^ RL: Reduced lighting regimen comprising intermittent 4 h light:2 h dark/24 h from 0–1 WOA, 18 h light/24 h from 1–2 WOA, 15 h light/24 h from 2–3 WOA, 12 h light/24 h from 3–4 WOA, and 9 h light/24 h from 4–16 WOA. ^4^ SEM: standard error of the mean. ^5^ Feeding: Feeding program for pullets between 4 and 16 WOA. ^6^ Ad lib: ad libitum feeding. ^7^ BSW: Breed standard weight feeding to achieve pullet breed standard weight for age from 4–16 WOA. ^8^ Managed: Feeding to achieve 88% pullet breed standard weight for age between 4 and 16 WOA. ^9^ Egg shape index: egg width divided by egg length ×100. ^10^ Yolk color score: DSM color fan, 1(palest) through to 15 (darkest) color scale. ^11^ Shell wt (%): shell weight as a percent of egg weight. ^12^ N: Newton. ^A–C^ Means within a column with different superscripts differ at *p* < 0.05.

## Data Availability

The original contributions in this study are included in the article; further inquiries can be directed to the corresponding author.
